# Association of Bone Mineral Density Testing With Risk of Major Osteoporotic Fractures Among Older Men Receiving Androgen Deprivation Therapy to Treat Localized or Regional Prostate Cancer

**DOI:** 10.1001/jamanetworkopen.2022.5432

**Published:** 2022-04-01

**Authors:** Maria E. Suarez-Almazor, Xerxes Pundole, Gerardo Cabanillas, Xiudong Lei, Hui Zhao, Linda S. Elting, Maria A. Lopez-Olivo, Sharon H. Giordano

**Affiliations:** 1 Department of Health Services Research, University of Texas MD Anderson Cancer Center, Houston; 2 Now with Amgen Inc, Thousand Oaks, California; 3 Department of Internal Medicine, Pacific Hospital of the Valley, Serra Medical Group, Los Angeles, California; 4 Department of Breast Medical Oncology, University of Texas MD Anderson Cancer Center, Houston

## Abstract

**Importance:**

Bone health screening is recommended for patients with prostate cancer who are initiating treatment with androgen deprivation therapy (ADT); however, bone mineral density screening rates in the US and their association with fracture prevention are unknown.

**Objective:**

To assess dual-energy x-ray absorptiometry (DXA) screening rates and their association with fracture rates among older men with prostate cancer initiating treatment with androgen deprivation therapy.

**Design, Setting, and Participants:**

This retrospective nationwide population-based cohort study used data from the Surveillance, Epidemiology, and End Results database and the Texas Cancer Registry linked with Medicare claims. Participants comprised 54 953 men 66 years or older with prostate cancer diagnosed between January 2005 and December 2015 who initiated treatment with ADT. Data were censored at last enrollment in Medicare and analyzed from January 1 to September 30, 2021.

**Exposures:**

Dual-energy x-ray absorptiometry screening within 12 months before and 6 months after the first ADT claim.

**Main Outcomes and Measures:**

Frequencies of DXA screening and fracture (any fracture and major osteoporotic fracture) and overall survival were calculated. The association between DXA screening and fracture was evaluated using a multivariable Cox proportional hazards model with propensity score adjustment.

**Results:**

Among 54 953 men (median age, 74 years; range, 66-99 years) with prostate cancer, 4689 (8.5%) were Hispanic, 6075 (11.1%) were non-Hispanic Black, 41 453 (75.4%) were non-Hispanic White, and 2736 (5.0%) were of other races and/or ethnicities (including 121 [0.2%] who were American Indian or Alaska Native; 1347 [2.5%] who were Asian, Hawaiian, or Pacific Islander; and 1268 [2.3%] who were of unknown race/ethnicity). Only 4362 men (7.9%) received DXA screening. The DXA screening rate increased from 6.8% in 2005 to 8.4% in 2015. Lower screening rates were associated with being single (odds ratio [OR], 0.89; 95% CI, 0.81-0.97; *P* = .01) and non-Hispanic Black (OR, 0.80; 95% CI, 0.70-0.91; *P* < .001), living in small urban areas (OR, 0.77; 95% CI, 0.66-0.90; *P* = .001) and areas with lower educational levels (OR, 0.75; 95% CI, 0.67-0.83; *P* < .001), and receiving nonsteroidal androgens (OR, 0.57; 95% CI, 0.39-0.84; *P* = .004). Overall, 9365 patients (17.5%) developed fractures after initial receipt of ADT. The median time to first fracture was 31 months (IQR, 15-56 months). In the multivariable model with propensity score adjustment, DXA screening was not associated with fracture risk at any site (hazard ratio [HR], 0.96; 95% CI, 0.89-1.04; *P* = .32) among men without previous fractures before receipt of ADT. However, previous DXA screening was associated with a decreased risk of major fractures (HR, 0.91; 95% CI, 0.83-1.00; *P* = .05) after propensity score adjustment.

**Conclusions and Relevance:**

In this study, low DXA screening rates were observed among older men with localized or regional prostate cancer after initiation of treatment with ADT. Despite low rates of screening, evaluation of bone mineral density with a DXA scan was associated with lower risk of major fractures. These findings suggest that DXA screening is important for the prevention of major fractures among older men with prostate cancer and that implementation strategies are needed to adopt bone health screening guidelines in clinical practice.

## Key Points

**Question:**

What are the rates of dual-energy x-ray absorptiometry (DXA) screening to assess bone mineral density among older men with prostate cancer who are beginning treatment with androgen deprivation therapy, and what is their association with fracture development?

**Findings:**

In this cohort study of 54 953 older men with prostate cancer, 4362 men (7.9%) received DXA screening; 9365 men (17.5%) developed any fracture, and 4114 men (7.7%) developed a major osteoporotic fracture. Bone mineral density testing was significantly associated with a decreased risk of developing major osteoporotic fractures after adjustment for covariates.

**Meaning:**

This study’s findings support the clinical importance of performing DXA screening for major fracture prevention among older men with prostate cancer.

## Teaser

This cohort study uses Surveillance, Epidemiology, and End Results; Texas Cancer Registry; and Medicare claims data to assess bone mineral density screening rates and their association with fracture rates among older men with prostate cancer who began treatment with androgen deprivation therapy.

## Introduction

Prostate cancer accounts for 26% of all new cancers in men.^1^ In 2021, an estimated 248 530 new cases of prostate cancer were projected to occur in the US.^1^ Androgen deprivation therapy (ADT) is the mainstay for treatment of locally advanced high-risk localized or metastatic prostate cancer.^2,3,4,5^ However, ADT can negatively impact bone health, resulting in decreased bone mineral density (BMD) and fractures.^6^ A high prevalence of osteoporosis (35.4%) was reported in hormone-naive men with prostate cancer, which increased to 80.6% after 10 years of ADT receipt.^7^

A study of men with prostate cancer using data from the Surveillance, Epidemiology, and End Results (SEER)–linked Medicare database between 1992 and 1997 reported that 19.4% of patients who received ADT developed a fracture between 12 and 60 months after diagnosis compared with 12.6% of patients who did not.^8^ A limitation of this study was that fractures associated with bone metastases were not excluded because the cohort included men with metastatic disease. A subsequent report of SEER-Medicare data from 1996 to 2003 found persistent increased fracture rates in this population, with mortality 2-fold higher among men who developed a fracture compared with those who did not.^9^ Similar increased mortality was observed in another study.^10^ Others have confirmed an increased fracture risk after receipt of ADT.^11,12,13^

Several systematic reviews from the early 2000s that reported on the risk of fracture with the use of ADT among men with prostate cancer as well as more recent guidelines from professional organizations advocate screening for osteoporosis using dual-energy x-ray absorptiometry (DXA).^14,15,16,17,18,19,20,21^ Yet screening rates remain low. A previous study using Texas Cancer Registry (TCR) and Medicare data found that only 8.6% of men initiating treatment with ADT received DXA screening.^22^ Other studies using data from the SEER-Medicare database,^23,24^ the Veterans Health Administration,^25^ and Canadian administrative claims^26,27^ reported similarly low rates (4.8%-17.8%).

Given the increased fracture risk and recommendations for BMD screening, we hypothesized that practice patterns and fracture risk among men with prostate cancer initiating treatment with ADT may have improved. To our knowledge, only 2 population-based studies^25,27^ have evaluated BMD screening among men initiating treatment with ADT; 1 study^27^ used the Quebec public health care insurance database, and the other^25^ used the Veterans Health Administration database. Thus, we conducted a larger national study using data from the SEER and TCR Medicare-linked databases to assess DXA screening and fracture rates among men 66 years or older with localized or regional prostate cancer who were initiating treatment with ADT.

## Methods

The institutional review board of the University of Texas MD Anderson Cancer Center approved this study and deemed it exempt from the need for informed consent because only deidentified data were used in the analyses. This study followed the Reporting of Studies Conducted Using Observational Routinely Collected Health Data Statement for Pharmacoepidemiology (RECORD-PE) reporting guideline.

### Data Sources

We used the SEER and TCR registries linked to Medicare claims.^28,29^ The SEER database includes data from 18 state cancer registries, representing 28% of the US population. The TCR is one of the largest statewide cancer registries (not included in the SEER database). Medicare is a national program that provides health insurance for patients 65 years or older in the US. Medicare claims for Part A (inpatient services) and Part B (outpatient services) were available for 2005 to 2016, and claims for Part D (pharmacy services) were available for 2007.

### Cohort

We identified men 66 years and older who had histologically confirmed prostate cancer diagnosed between January 1, 2005, and December 31, 2015. Data were censored at last enrollment in Medicare and analyzed from January 1 to September 30, 2021. Inclusion criteria included (1) prostate cancer classified by the SEER registry as localized (limited to the organ of origin with no spread beyond the organ of origin) or regional (extended beyond the limits of the organ of origin); (2) receipt of orchiectomy or ADT between 30 days before diagnosis (to control for delays in cancer diagnosis registration) and 1 year after diagnosis, identified using procedure codes from the *International Classification of Diseases, Ninth Revision, Clinical Modification*; the *International Classification of Diseases, Tenth Revision, Clinical Modification;* or the Healthcare Common Procedure Coding System (eTable 1 in the Supplement shows parenteral and oral ADT for men enrolled in Medicare Part D and parenteral ADT alone for men without Part D coverage); (3) no coverage by a health maintenance organization for at least 12 months before and 12 months after the first ADT claim; and (4) survival at least 1 year after ADT initiation. Exclusion criteria included (1) distant or metastatic cancer (spread to areas beyond primary tumor) to avoid misclassification of bone fractures associated with metastasis and (2) fracture in the same anatomical location within the 12 months before and 12 months after the first ADT claim to avoid including patients with follow-up claims for a fracture that occurred before ADT initiation.

### Outcomes of Interest

The primary outcomes of interest were the frequencies of DXA screening and fracture (any fracture and major osteoporotic fracture) and overall survival (OS). The frequency of DXA screening (Healthcare Common Procedure codes 76075 and 77080) was examined from 12 months before to 6 months after the initial ADT claim. We included DXA screening before ADT initiation because Medicare reimburses DXA screening every 24 months for preventive care or every 12 months for patients at risk of fracture. Therefore, if a man had received a DXA screening within the 12 months before ADT initiation, this DXA would be appropriate as baseline screening because Medicare would not pay for another DXA at initiation of treatment with ADT.

Analyses of the frequency of any fracture or major osteoporotic fracture (spine, upper arm, lower arm, hip, and other femur) excluded men who experienced fractures in the 12 months before ADT initiation because they would have been more likely to receive DXA screening as a consequence of the previous fracture (eTable 2 in the Supplement). We also identified the use of bone-modifying agents (ie, bisphosphonates, denosumab, and teriparatide) among the subset of enrollees with Medicare Part D using either generic names from Part D files or Healthcare Common Procedure codes from Part A and Part B files (eTable 3 in the Supplement). Overall survival (OS) was estimated from the date of prostate cancer diagnosis to the date of death, with censoring at last follow-up.

### Covariates

Demographic variables included age, race and ethnicity (as recorded in databases; known differences in bone density and fracture rates exist across racial and ethnic groups), marital status, area of residence, state buy-in status (state buy-ins are programs that assist with the cost of Medicare; receiving this aid was used as a proxy for low socioeconomic status), and state of registry. Surrogate values for educational level (in quartiles, with 1 indicating highest and 4 indicating lowest) and poverty level (in quartiles, with 1 indicating lowest and 4 indicating highest) were assigned on the basis of median levels within the participant’s 2000 US census tract.

Tumor-related variables included cancer stage (localized or regional), cancer grade (defined by SEER categories into low [grades I-II] or high [grades III-IV]), year of ADT initiation, and ADT type. Previous osteoporosis and fractures were ascertained within 12 months before the initial ADT claim. Comorbidities present in the year before cancer diagnosis were estimated using Charlson Comorbidity Index scores (categorized as 0, 1, and ≥2). Cancer was excluded as a comorbidity.^30,31^

### Statistical Analysis

We estimated DXA screening rates and 95% CIs assuming a binomial distribution. To identify factors associated with DXA screening, we used χ^2^ tests for nominal variables and the Cochrane-Armitage trend test for ordinal categorical variables. We built a multivariable logistic regression model for DXA screening that was fit with covariates using a backward reduction selection method. Variables with a statistical significance of *P* ≤ .05 were retained. The final model included year of initial ADT (2005-2015), cancer registry (SEER state registry), age (66-70 years, 71-75 years, 76-80 years, or >80 years), marital status (single vs married), race and ethnicity (Hispanic, non-Hispanic Black, non-Hispanic White, or other [including American Indian or Alaska Native; Asian, Hawaiian, or Pacific Islander; or unknown]), cancer stage (localized vs regional), cancer grade (low vs high), Charlson comorbidity score (0, 1, or ≥2), ADT type (leuprolide only, goserelin only, triptorelin only, abarelix or degarelix only, histrelin only, nonsteroidal antiandrogen, or ≥2 ADT types), state buy-in status (none vs full or partial), and previous osteoporosis (yes vs no). Based on this model, we generated a propensity score for being in the DXA group, which was then used as a covariate in multivariable models.

We defined time to first fracture as the time from the first ADT claim to the date of first fracture, censoring at the date of death or the last day of Medicare coverage, whichever came first. The last year of available Medicare claims was 2016. We evaluated any fracture, major fractures, and each fracture site. All fractures occurred after the initial DXA claim. We plotted Kaplan-Meier curves for time to first fracture. To adjust for selection bias between cohorts who did and did not receive DXA screening, we conducted a Cox proportional hazards multivariable analysis for time to fracture, adjusted for propensity score. We conducted sensitivity analyses including only fractures that occurred after 12 months of ADT.

We estimated OS from initial receipt of ADT until death (event) or last follow-up (censor), plotting Kaplan-Meier curves stratified by fracture and comparing groups using log rank tests. We ascertained the use of bone-modifying agents among patients who had DXA claims from the date of the initial DXA screening to 6 months after the screening; for those without DXA claims, we used the date of initial receipt of ADT to 6 months after receipt.

Statistical significance was set at 2-tailed *P* < .05. Analyses were performed using SAS software, version 9.3 (SAS Institute Inc).

## Results

Among 54 953 patients, 47 270 (86.0%) were identified from the SEER database and 7683 (14.0%) from the TCR (eFigure in the Supplement). The median age was 74 years (range, 66-99 years), and the median time from cancer diagnosis to initial receipt of ADT was 34 days (IQR, 19-61 days). A total of 4689 patients (8.5%) were Hispanic, 6075 (11.1%) were non-Hispanic Black, 41 453 (75.4%) were non-Hispanic White, and 2736 (5.0%) were of other races and/or ethnicities (including 121 [0.2%] who were American Indian or Alaska Native; 1347 [2.5%] who were Asian, Hawaiian, or Pacific Islander; and 1268 [2.3%] who were of unknown race/ethnicity). Most patients had localized disease (42 293 men [89.7%]) that was high grade (38 782 men [70.6%]). Leuprolide, a gonadotropin-releasing hormone agonist, was the most commonly used type of ADT (24 719 men [45.0%]); 21 512 men (39.2%) received 2 or more types of ADT within the first year after diagnosis. Only 53 patients (0.1%) received orchiectomy. Overall, 4362 men (7.9%; 95% CI, 7.7%-8.2%) received DXA screening. Between 2005 and 2015, the DXA screening rate increased from 6.8% to 8.4% (*P* = .03). Non-Hispanic Black patients had the lowest DXA screening rate (5.2%). Additional demographic and clinical characteristics of the entire cohort and the men who received DXA screening are shown in Table 1.

**Table 1.** Demographic and Tumor Characteristics of Patients Who Received DXA Screening^a^

Characteristic	Total patients, No.	Patients who received DXA screening, No. (%)	*P* value^b^
Total participants	54 953	4362 (7.9)	NA
Year of initial ADT			
2005	6035	412 (6.8)	.03
2006	7183	539 (7.5)	
2007	7343	591 (8.0)	
2008	5860	478 (8.2)	
2009	4998	432 (8.6)	
2010	4754	389 (8.2)	
2011	4836	403 (8.3)	
2012	3814	307 (8.0)	
2013	3534	283 (8.0)	
2014	2967	222 (7.5)	
2015	3629	306 (8.4)	
SEER state registry			
California	13 116	1233 (9.4)	<.001
Connecticut	2716	235 (8.7)	
Detroit	2951	266 (9.0)	
Georgia	4678	213 (4.6)	
Hawaii	715	96 (13.4)	
Iowa	2984	126 (4.2)	
Kentucky	2996	179 (6.0)	
Louisiana	3881	193 (5.0)	
New Jersey	8658	698 (8.1)	
New Mexico	958	112 (11.7)	
Seattle	2424	165 (6.8)	
Texas	7683	781 (10.2)	
Utah	1193	65 (5.4)	
Age, y			
Median (range)	74 (66-99)	NA	NA
66-70	12 505	784 (6.3)	<.001
71-75	16 852	1231 (7.3)	
76-80	14 169	1236 (8.7)	
>80	11 427	1111 (9.7)	
Marital status			
Married	33 058	2603 (7.9)	<.001
Single	10 613	739 (7.0)	
Unknown	11 282	1020 (9.0)	
Race and ethnicity			
Hispanic	4689	393 (8.4)	<.001
Non-Hispanic Black	6075	317 (5.2)	
Non-Hispanic White	41 453	3341 (8.1)	
Other^c^	2736	311 (11.4)	
Cancer stage			
Localized	49 293	3739 (7.6)	<.001
Regional	5660	623 (11.0)	
Cancer grade			
Low	14 396	901 (6.3)	<.001
High	38 782	3270 (8.4)	
Unknown	1775	191 (10.8)	
Charlson Comorbidity Index score			
0	31 136	2264 (7.3)	<.001
1	13 662	1133 (8.3)	
≥2	9826	951 (9.7)	
Unknown	329	14 (4.3)	
ADT type			
Leuprolide	24 719	1762 (7.1)	<.001
Goserelin	3536	232 (6.6)	
Triptorelin	3597	222 (6.2)	
Abarelix or degarelix	443	43 (9.7)	
Histrelin	406	34 (8.4)	
Nonsteroidal antiandrogen	687	32 (4.7)	
≥2 ADT types	21 512	2037 (9.5)	
Area of residence^d^			
Big metropolitan	28 106	2387 (8.5)	<.001
Metropolitan	16 807	1393 (8.3)	
Urban	3439	239 (6.9)	
Small urban	5410	273 (5.0)	
Rural	1191	70 (5.9)	
State buy-in status			
None	48 455	3874 (8.0)	.17
Full or partial	6498	488 (7.5)	
Educational level, quartile^e^			
1	12 851	1249 (9.7)	<.001
2	12 841	1026 (8.0)	
3	13 344	960 (7.2)	
4	13 926	938 (6.7)	
Unknown	1991	189 (9.5)	
Poverty level, quartile^f^			
1	12 692	1225 (9.7)	<.001
2	12 858	1060 (8.2)	
3	13 514	924 (6.8)	
4	13 898	964 (6.9)	
Unknown	1991	189 (9.5)	
Osteoporosis before first ADT or DXA			
No	54 427	4012 (7.4)	<.001
Yes	1526	350 (22.9)	
Fracture before first ADT			
No	53 527	4169 (7.8)	<.001
Yes	1426	193 (13.5)	

Abbreviations: ADT, androgen deprivation therapy; DXA, dual-energy x-ray absorptiometry; NA, not applicable; SEER, Surveillance, Epidemiology, and End Results.

^a^ A total of 53 participants (0.1%) received orchiectomy. Exact values were not presented to adhere to the current policy of the Centers for Medicare and Medicaid Services, which recommends avoiding publication of table cells containing 10 or fewer individuals to protect patient anonymity.

^b^ *P* values from χ^2^ test or trend test if indicated.

^c^ Other category included 121 participants (0.2%) who were American Indian or Alaska Native; 1347 (2.5%) who were Asian, Hawaiian, or Pacific Islander; and 1268 (2.3%) who were of unknown race and/or ethnicity.

^d^ Big metropolitan refers to counties in metropolitan areas with population of 1 million or more; metropolitan refers to counties in metropolitan areas with population of 250 000 or fewer to 1 million; urban refers to population of 20 000 or more; small urban refers to population of 2500 to 19 999; rural refers to population fewer than 2500.

^e^ Quartiles were based on the median educational level within the participant’s 2000 US census tract. Quartile 1 indicates highest educational level (ie, most likely to have a high school diploma), and quartile 4 indicates lowest educational level.

^f^ Quartiles were based on the median poverty level within the participant’s 2000 US census tract. Quartile 1 indicates lowest poverty level, and quartile 4 indicates highest poverty level.

### Factors Associated With DXA Screening

In the multivariable model (Table 2), non-Hispanic Black patients were 20% less likely (odds ratio [OR], 0.80; 95% CI, 0.70-0.91; *P* < .001) to receive DXA screening than non-Hispanic White patients. Single status (OR, 0.89; 95% CI, 0.81-0.97; *P* = .01), full or partial state buy-in (OR, 0.78; 95% CI, 0.70-0.87; *P* < .001), living in a small urban area (OR, 0.77; 95% CI, 0.66-0.90; *P* = .001) or an area with a lower educational level (eg, quartile 4: OR, 0.75; 95% CI, 0.67-0.83; *P* < .001), and receipt of nonsteroidal antiandrogens (OR, 0.57; 95% CI, 0.39-0.84; *P* = .004) were also associated with lower DXA screening rates. Higher DXA screening rates were associated with recent year of ADT initiation (2015: OR, 1.44; 95% CI, 1.20-1.71; *P* < .001), state of residence (eg, New Mexico: OR, 1.59 [95% CI, 1.26-2.00; *P* < .001]; Texas: OR, 1.22 [95% CI, 1.08-1.37; *P* < .001]), age 76 years and older (76-80 years: OR, 1.27 [95% CI, 1.15-1.41; *P* < .001]; >80 years: OR, 1.25 [95% CI, 1.12-1.39; *P* < .001]), regional disease (OR, 1.44; 95% CI, 1.30-1.59; *P* < .001), high-grade disease (OR, 1.25; 95% CI, 1.15-1.37; *P* < .001), Charlson comorbidity score of 1 (OR, 1.09; 95% CI, 1.00-1.18 *P* = .047) to 2 or higher (OR, 1.25; 95% CI, 1.14-1.36; *P* < .001), receipt of the gonadotropin-releasing hormone agonists abarelix or degarelix (OR, 1.42; 95% CI, 1.01-2.00; *P* = .046) or multiple types of ADT (OR, 1.38; 95% CI, 1.28-1.49; *P* < .001), previous fracture (OR, 1.21; 95% CI, 1.01-1.45; *P* = .03), and presence of osteoporosis before initiation of ADT (OR, 16.02; 95% CI, 14.66-17.51; *P* < .001).

**Table 2.** Multivariable Logistic Regression Model for Association Between Participant Characteristics and DXA Screening^a^

Covariate^a^	OR (95% CI)	*P* value
Year of initial ADT		
2005	1 [Reference]	NA
2006	1.09 (0.94-1.27)	.24
2007	1.21 (1.04-1.41)	.01
2008	1.22 (1.04-1.42)	.02
2009	1.27 (1.08-1.50)	.003
2010	1.25 (1.06-1.47)	.008
2011	1.19 (1.01-1.40)	.04
2012	1.10 (0.92-1.31)	.29
2013	1.13 (0.95-1.35)	.18
2014	1.18 (0.97-1.42)	.09
2015	1.44 (1.20-1.71)	<.001
SEER state registry		
California	1 [Reference]	NA
Connecticut	0.99 (0.84-1.16)	.91
Detroit	1.03 (0.88-1.21)	.68
Georgia	0.65 (0.55-0.76)	<.001
Hawaii	1.30 (0.99-1.70)	.06
Iowa	0.52 (0.42-0.64)	<.001
Kentucky	0.81 (0.67-0.97)	.02
Louisiana	0.73 (0.62-0.87)	<.001
New Jersey	0.85 (0.76-0.95)	.004
New Mexico	1.59 (1.26-2.00)	<.001
Seattle	0.81 (0.67-0.97)	.02
Texas	1.22 (1.08-1.37)	<.001
Utah	0.64 (0.49-0.84)	.001
Age, y		
66-70	1 [Reference]	NA
71-75	1.09 (0.99-1.21)	.07
76-80	1.27 (1.15-1.41)	<.001
>80	1.25 (1.12-1.39)	<.001
Marital status		
Married	1 [Reference]	NA
Single	0.89 (0.81-0.97)	.01
Race and ethnicity		
Hispanic	0.91 (0.79-1.04)	.16
Non-Hispanic Black	0.80 (0.70-0.91)	<.001
Non-Hispanic White	1 [Reference]	NA
Other^b^	1.10 (0.94-1.29)	.25
Stage		
Localized	1 [Reference]	NA
Regional	1.44 (1.30-1.59)	<.001
Grade		
Low	1 [Reference]	NA
High	1.25 (1.15-1.37)	<.001
Charlson Comorbidity Index score		
0	1 [Reference]	NA
1	1.09 (1.00-1.18)	.047
≥2	1.25 (1.14-1.36)	<.001
ADT type		
Leuprolide only	1 [Reference]	NA
Goserelin only	1.15 (0.97-1.35)	.11
Triptorelin only	0.92 (0.78-1.07)	.28
Abarelix or degarelix only	1.42 (1.01-2.00)	.046
Histrelin only	1.11 (0.75-1.63)	.60
Nonsteroidal antiandrogen	0.57 (0.39-0.84)	.004
≥2 ADT types	1.38 (1.28-1.49)	<.001
Residence area^c^		
Big metropolitan	1 [Reference]	NA
Metropolitan	1.12 (1.03-1.22)	.008
Urban	0.94 (0.81-1.10)	.47
Small urban	0.77 (0.66-0.90)	.001
Rural	1.01 (0.77-1.33)	.92
State buy-in status		
None	1 [Reference]	NA
Full or partial	0.78 (0.70-0.87)	<.001
Educational level, quartile^d^		
1	1 [Reference]	NA
2	0.88 (0.80-0.96)	.007
3	0.82 (0.74-0.90)	<.001
4	0.75 (0.67-0.83)	<.001
Osteoporosis before first ADT		
No	1 [Reference]	NA
Yes	16.02 (14.66-17.51)	<.001
Fracture before first ADT		
No	1 [Reference]	NA
Yes	1.21 (1.01-1.45)	.03

Abbreviations: ADT, androgen deprivation therapy; DXA, dual-energy x-ray absorptiometry; NA, not applicable; OR, odds ratio; SEER, Surveillance, Epidemiology, and End Results.

^a^ A total of 54 953 participants were included in the analysis.

^b^ Other races included American Indian or Alaska Native; Asian, Hawaiian, or Pacific Islander; or unknown.

^c^ Big metropolitan refers to counties in metropolitan areas with population of 1 million or more; metropolitan refers to counties in metropolitan areas with population of 250 000 or fewer to 1 million; urban refers to population of 20 000 or more; small urban refers to population of 2500 to 19 999; rural refers to population fewer than 2500.

^d^ Quartiles were based on the median educational level within the participant’s 2000 US census tract. Quartile 1 indicates highest educational level (ie, most likely to have a high school diploma), and quartile 4 indicates lowest educational level.

### Association of DXA Screening With Fracture

For these analyses, we identified 53 472 men who had no fracture within the 12 months before ADT initiation. The median follow-up was 58 months (IQR, 32-92 months). A total of 9365 patients (17.5%) developed a fracture at any site, and 4114 patients (7.7%) developed a major fracture, with a median time to first fracture of 31 months (IQR, 15-56 months). The 5-year estimate was 84% for any fracture-free survival and 89% for major fracture–free survival. Among 4114 patients who received DXA screening, 830 men (20.2%) developed a fracture at any site compared with 8535 of 49 358 men (17.3%; *P* < .001) who did not receive DXA screening. Major fractures were observed in 612 men (14.9%) who received DXA screening compared with 6438 men (13.0%; *P* < .001) who did not. Fractures at various anatomical sites based on DXA screening status are shown in eTable 4 in the Supplement.

In the multivariable model including propensity score adjustment, previous DXA screening was not significantly associated with the risk of any fracture (hazard ratio [HR], 0.96; 95% CI, 0.89-1.04; *P* = .32) (eTable 5 in the Supplement). However, previous DXA screening was associated with a 9.1% decrease in the risk of major fractures (HR, 0.91; 95% CI, 0.83-1.00; *P* = .05) after adjustment (Table 3). An increased risk of both any fracture and major fracture within 12 months before ADT initiation was associated with earlier year of treatment initiation (eg, any fracture in 2008: HR, 0.81 [95% CI, 0.75-0.88; *P* < .001]; major fracture in 2008: HR, 0.88 [95% CI, 0.80-0.97; *P* = .008]), older age (eg, any fracture at >80 years: HR, 2.18 [95% CI, 2.04-2.33; *P* < .001]; major fracture at >80 years: HR, 2.83 [95% CI, 2.62-3.06; *P* < .001]), single status (any fracture: HR, 1.16 [95% CI, 1.10-1.23; *P* < .001]; major fracture: HR, 1.18 [95% CI, 1.11-1.26; *P* < .001]), non-Hispanic White race and ethnicity (HR, 1.00 [reference variable] for any and major fracture), regional cancer (any fracture: HR, 1.17 [95% CI, 1.08-1.26; *P* < .001]; major fracture: HR, 1.16 [95% CI, 1.06-1.27; *P* < .001]), high-grade cancer (any fracture: HR, 1.11 [95% CI, 1.05-1.17; *P* < .001]; major fracture: HR, 1.18 [95% CI, 1.11-1.25; *P* < .001]), more comorbidities (any fracture with ≥2 comorbidities: HR, 1.53 [95% CI, 1.45-1.62; *P* < .001]; major fracture with ≥2 comorbidities: HR, 1.55 [95% CI, 1.46-1.65; *P* < .001]), and full or partial state buy-in (any fracture: HR, 1.15 [95% CI, 1.08-1.23; *P* < .001]; major fracture: HR, 1.25 [95% CI, 1.16-1.34; *P* < .001]). The sensitivity analyses for major fractures occurring 12 months or more after initiation of ADT revealed similar patterns, but these patterns were not statistically significant (HR, 0.92; 95% CI, 0.84-1.01; *P* = .08).

**Table 3.** Propensity Score–Adjusted Multivariable Cox Proportional Hazards Model for Time to First Major Fracture After Initial ADT Among Patients With No Previous Fracture Within 12 Months Before Treatment Initiation^a^

Covariate	HR (95% CI)	*P* value
Receipt of DXA screening		
No	1 [Reference]	NA
Yes	0.91 (0.83-1.00)	.05
Year of initial ADT		
2005	1 [Reference]	NA
2006	0.99 (0.91-1.07)	.80
2007	0.96 (0.88-1.04)	.32
2008	0.88 (0.80-0.97)	.008
2009	0.83 (0.75-0.92)	<.001
2010	0.80 (0.71-0.89)	<.001
2011	0.74 (0.66-0.83)	<.001
2012	0.68 (0.60-0.77)	<.001
2013	0.63 (0.54-0.73)	<.001
2014	0.56 (0.47-0.67)	<.001
2015	0.38 (0.30-0.47)	<.001
SEER state registry		
California	1 [Reference]	NA
Connecticut	0.94 (0.83-1.05)	.28
Detroit	1.05 (0.94-1.18)	.38
Georgia	1.04 (0.94-1.16)	.46
Hawaii	1.12 (0.88-1.42)	.37
Iowa	0.97 (0.86-1.09)	.60
Kentucky	1.13 (1.00-1.26)	.04
Louisiana	1.10 (0.98-1.22)	.10
New Jersey	0.89 (0.83-0.97)	.007
New Mexico	1.13 (0.94-1.36)	.18
Seattle	1.00 (0.89-1.13)	.99
Texas	1.14 (1.04-1.24)	.004
Utah	0.92 (0.77-1.10)	.38
Age, y		
66-70	1 [Reference]	NA
71-75	1.22 (1.13-1.32)	<.001
76-80	1.79 (1.66-1.94)	<.001
>80	2.83 (2.62-3.06)	<.001
Marital status		
Married	1 [Reference]	NA
Single	1.18 (1.11-1.26)	<.001
Race and ethnicity		
Hispanic	0.72 (0.65-0.80)	<.001
Non-Hispanic Black	0.50 (0.45-0.56)	<.001
Non-Hispanic White	1 [Reference]	NA
Other^b^	0.50 (0.44-0.58)	<.001
Stage		
Localized	1 [Reference]	NA
Regional	1.16 (1.06-1.27)	<.001
Grade		
Low	1 [Reference]	NA
High	1.18 (1.11-1.25)	<.001
Charlson Comorbidity Index score		
0	1 [Reference]	NA
1	1.22 (1.15-1.29)	<.001
≥2	1.55 (1.46-1.65)	<.001
ADT type		
Leuprolide only	1 [Reference]	NA
Goserelin only	0.93 (0.84-1.03)	.15
Triptorelin only	0.89 (0.80-0.99)	.03
Abarelix or degarelix only	0.98 (0.68-1.41)	.93
Histrelin only	1.22 (0.96-1.55)	.11
Nonsteroidal antiandrogen	0.87 (0.66-1.15)	.33
≥2 ADT types	1.08 (1.02-1.15)	.01
State buy-in status		
None	1 [Reference]	NA
Full or partial	1.25 (1.16-1.34)	<.001
Osteoporosis before initial ADT		
No	1 [Reference]	NA
Yes	1.17 (0.63-2.15)	.62

Abbreviations: ADT, androgen deprivation therapy; DXA, dual-energy x-ray absorptiometry; HR, hazard ratio; NA, not applicable; SEER, Surveillance, Epidemiology, and End Results.

^a^ A total of 53 472 participants were included in the analysis. All participants with previous fractures or fractures before their last DXA claim were excluded. DXA screening was forced into the multivariable model. Adjusted variables remained in the model based on both clinical and statistical significance.

^b^ Other races included American Indian or Alaska Native; Asian, Hawaiian, or Pacific Islander; or unknown.

In total, there were 20 954 deaths (39.2%) among those who had no fractures within the 12 months before ADT initiation, and the median OS was 9.6 years (IQR, 3.5-9.0 years). The unadjusted 5-year OS estimates were 78% among men who did not develop a fracture, 77% among those who developed a fracture at any site (Figure, A), and 74% among men who developed a major fracture (*P* < .001 for both comparisons) (Figure, B).

**Figure.**
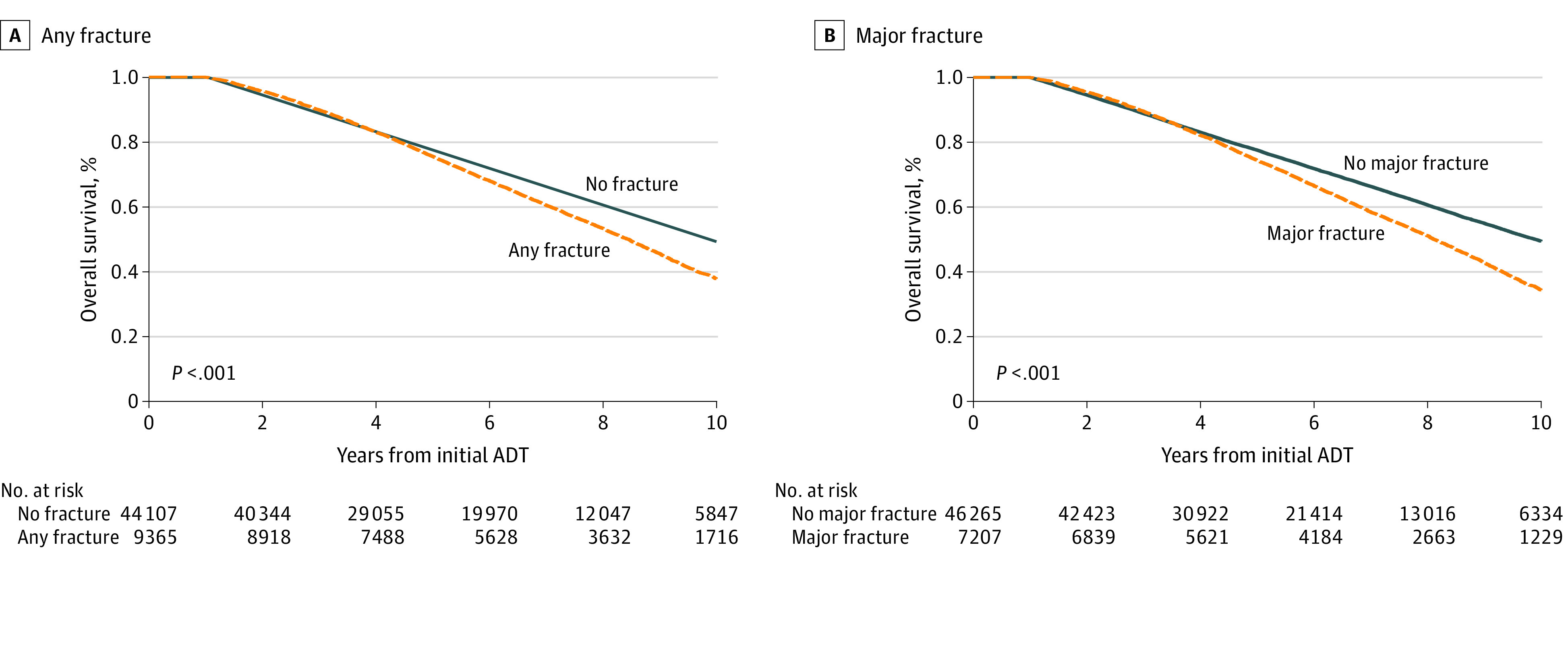
Overall Survival Among Participants With and Without Fractures ADT indicates androgen deprivation therapy.

### Association of DXA Screening With Bone-Modifying Agents

A total of 27 402 patients (49.9%) had Medicare Part D coverage for 6 months after initial receipt of ADT. Of those, 854 patients (3.1%) received bone-modifying agents (Table 4). Use of bone-modifying agents was higher among those who received DXA screening (401 of 2136 patients [18.8%]) vs those who did not (453 of 25 266 patients [1.8%]; *P* < .001). Among patients who received a bone-modifying agent, most received bisphosphonate alone (761 patients [89.1%]), followed by denosumab with and without another drug (94 patients [11.0%]). Most patients (831 [97.3%]) received a single drug.

**Table 4.** Association of DXA Screening With Receipt of Bone-Modifying Agent

Type of bone-conserving agent	No. (%)			*P* value
	All patients	Receipt of DXA screening		
		No	Yes	
Total participants, No.	27 402	25 266	2136	NA
Any^a^				
No	26 548 (96.9)	24 813 (98.2)	1735 (81.2)	<.001
Yes	854 (3.1)	453 (1.8)	401 (18.8)	
Bisphosphonate				
No	26 641 (97.2)	24 876 (98.5)	1765 (82.6)	<.001
Yes	761 (2.8)	390 (1.5)	371 (17.4)	
Denosumab				
No	27 308 (99.7)	25 201 (99.7)	2107 (98.6)	<.001
Yes	94 (0.3)	65 (0.3)	29 (1.4)	

Abbreviations: DXA, dual-energy x-ray absorptiometry; NA, not applicable.

^a^ Analysis included participants with Medicare Part D coverage for 6 months after initiation of ADT. Among participants who received DXA screening, treatment for osteoporosis was assessed from DXA screening date to 6 months after DXA screening date. Among participants who did not receive DXA screening, treatment for osteoporosis was counted from the date of the initial ADT claim to 6 months after treatment with ADT was completed. Exact values for participants receiving teriparitide were not presented to adhere to the current policy of the Centers for Medicare and Medicaid Services, which recommends avoiding publication of table cells containing 10 or fewer individuals to protect patient anonymity.

## Discussion

This cohort study found low nationwide rates (7.9%) of DXA screening (the standard of care for BMD assessment among patients with a high risk of osteoporosis^2,14,19,20,21,32^) among older men with localized or regional prostate cancer who initiated ADT, with small increases from 6.8% in 2005 to 8.4% in 2015. Factors associated with receipt of DXA screening included older age, history of osteoporosis or fractures, more advanced or high-risk cancer, and a greater number of comorbidities. A Veterans Health Administration study^25^ of 17 017 men with prostate cancer who received ADT reported a slightly higher screening rate of 15%, with higher rates among older patients and those with high-risk cancer. Other studies^22,23,24,25,27,33,34^ have also reported low DXA screening rates in this population, ranging from 4.8% to 17.8%, with only 1 small study^34^ of 149 men in Ontario, Canada, reporting a baseline DXA screening rate of 58.8%. These studies confirm the care gap that exists in bone health management and fracture prevention in this high-risk population.

Despite all men having access to Medicare insurance that covered the use of DXA screening, we identified demographic and socioeconomic disparities, finding lower DXA screening rates among men who were non-Hispanic Black or single, had state buy-in status (as a surrogate of poverty), and lived in census tract areas with lower educational levels and higher poverty levels. Differences across states were also observed.

Men with prostate cancer initiating treatment with ADT are at high risk of bone loss and fractures. A previous study^8^ of SEER-Medicare data involving men diagnosed with prostate cancer between 1992 and 1997 reported a rate of 19.37% for any fracture. Our study excluded patients with metastatic disease at diagnosis, and we did not require a minimum survival of 60 months, as specified in that study.^8^ However, fracture rates in our study remained high, with 17.5% of men developing a fracture at any site (50% of whom developed a fracture within 31 months) and 7.7% of men developing a major fracture. We found several factors associated with time to first fracture, including older age, single status, regional disease stage, high cancer grade, higher Charlson comorbidity score, full or partial state buy-in status, and receipt of 2 or more types of ADT. Use of DXA screening was more prevalent among patients with a diagnosis of osteoporosis or fractures within the 12 months before initiation of treatment with ADT. However, after excluding patients with previous fracture and adjusting for previous osteoporosis in the multivariable model, we found a modest but statistically significant 9.1% lower risk of major fractures among men who received DXA screening compared with those who did not. No statistically significant association was observed between DXA screening and fracture at any site. However, because few patients received DXA screening, a potential benefit may have been missed.

Two studies,^35,36^ including a recent systematic review,^36^ have reported that receipt of bisphosphonates and denosumab were associated with reduced bone loss among patients with prostate cancer. Given that therapies to prevent bone loss already exist, the use of DXA screening to identify patients with an indication for therapy is important for adequate bone health management. Few men (3.1%) received bone-modifying agents in our study. Men who received DXA screening were more likely to receive bone-modifying agents (18.8%) than those who did not (1.8%). We were not able to ascertain the association between bone-modifying agents and fracture incidence because only a small subset of patients received these medications. A previous study^37^ reported similar results among women with breast cancer who initiated treatment with aromatase inhibitors, as identified in the SEER and TCR Medicare-linked databases. In that study, DXA screening was also associated with a 9% decrease in the risk of fractures and a higher likelihood of receiving bone-modifying agents.^37^

Notably, fractures in older individuals have been associated with prolonged immobilization and high morbidity and mortality, especially when these fractures occur at major anatomical sites.^38,39,40,41,42^ In our study, mortality was also higher, with a 5-year OS of 74% among men who had a major fracture compared with 78% among men without fractures (*P* < .001).

To our knowledge, this study conducted the largest population-based analysis to date of DXA screening rates among men with prostate cancer who initiated ADT. The linking of large cancer registries with administrative claims provided a rich source of data to evaluate outcomes among patients with cancer. Cancer is histologically confirmed in cancer registries, which reduces misclassification of cancer when using administrative claims data alone.

### Limitations

This study has several limitations. Although we included patients with early disease (unlike other studies that included patients with distant metastases), we used claims data, so we cannot entirely eliminate the possibility that some fractures were metastatic rather than osteoporotic. In addition, most patients (89.7%) had localized disease, limiting the generalizability of our results. However, 90% of prostate cancer cases are discovered at early stages (localized or regional)^43^; hence, our results are applicable to most patients seen in clinical practice. Our analysis is subject to the inherent limitations of observational claims research and, although we controlled for several potential confounders in our multivariable models, we could not fully account for unobserved confounders. We evaluated patients older than 65 years; thus, results are not generalizable to younger patients. However, 60% of prostate cancer cases are diagnosed in men older than 65 years,^44^ and our results are applicable to this population. In addition, we used Charlson scores to evaluate comorbidities, but this index may be inadequate to evaluate risk factors associated with fractures.

## Conclusions

In this cohort study, the use of DXA screening among older men with localized or regional prostate cancer who initiated ADT remained low and was associated with racial, socioeconomic, and geographic disparities. The use of DXA screening shortly before or after the initiation of ADT was associated with a reduction in major fractures. Given the deleterious impact of fractures for morbidity and mortality, implementation strategies are needed to increase the uptake of current guidelines for bone health management among men with prostate cancer. Early intervention with bone-modifying agents could potentially reduce the burden of illness associated with fractures among older men who are survivors of prostate cancer.

## Notes

Supplement. **eTable 1.** Codes Used to Identify ADT, DXA Screening, and Osteoporosis **eTable 2.** Codes Used to Identify Fractures **eTable 3.** List of HCPCS Codes Used to Identify Bone-Modifying Agents **eTable 4.** Proportions of Patients With Fractures After Initial ADT According to DXA Screening **eTable 5.** Multivariable Cox Proportional Hazards Model for Time to First Fracture at Any Site After Initial ADT With Adjustment for Propensity Scores **eFigure.** Data Sources and Cohort Selection
