# Supplementary material for: Association of Bone Mineral Density Testing With Risk of Major Osteoporotic Fractures Among Older Men Receiving Androgen Deprivation Therapy to Treat Localized or Regional Prostate Cancer
Source: JAMA Netw Open. 2022 Apr 1;5(4):e225432. doi: 10.1001/jamanetworkopen.2022.5432 (PMC8976238; doi:10.1001/jamanetworkopen.2022.5432)
Supplement: Supplement. — eTable 1. Codes Used to Identify ADT, DXA Screening, and Osteoporosis eTable 2. Codes Used to Identify Fractures eTable 3. List of HCPCS Codes Used to Identify Bone-Modifying Agents eTable 4. Proportions of Patients With Fractures After Initial ADT According to DXA Screening eTable 5. Multivariable Cox Proportional Hazards Model for Time to First Fracture at Any Site After Initial ADT With Adjustment for Propensity Scores eFigure. Data Sources and Cohort Selection [file jamanetwopen-e225432-s001.pdf]

Supplementary Online Content

Suarez-Almazor ME, Pundole X, Cabanillas G, et al. Association of bone mineral density testing with risk of major osteoporotic fractures among older men receiving androgen deprivation therapy to treat localized or regional prostate cancer. *JAMA Netw Open*. 2022;5(4):e225432. doi:10.1001/jamanetworkopen.2022.5432

eTable 1. Codes Used to Identify ADT, DXA Screening, and Osteoporosis

eTable 2. Codes Used to Identify Fractures

eTable 3. List of HCPCS Codes Used to Identify Bone-Modifying Agents

eTable 4. Proportions of Patients With Fractures After Initial ADT According to DXA Screening

eTable 5. Multivariable Cox Proportional Hazards Model for Time to First Fracture at Any Site After Initial ADT With Adjustment for Propensity Scores

eFigure. Data Sources and Cohort Selection

This supplementary material has been provided by the authors to give readers additional information about their work.

© 2022 Suarez-Almazor ME et al. *JAMA Network Open*.

eTable 1. Codes Used to Identify ADT, DXA Screening, and Osteoporosis

**Description**	**ICD-9 or ICD-10 Diagnosis Codes**	**ICD-9 or ICD-10 Procedure Codes**	**HCPCS Codes**
ADT			J0128, J1675, J1950, J3315, J9155, J9202, J9217, J9218, J9219, J9225, J9226
Orchiectomy		(ICD-9) 62.4, 62.41, 62.42; (ICD-10) OVTC0ZZ, OVTC4ZZ, OVT90ZZ, OVT94ZZ, OVTB0ZZ, OVTB4ZZ	54520, 54521, 54522, 54530, 54535
DXA screening			76075, 77080
Osteoporosis	(ICD-9) 733.0, 733.00, 733.02, 733.03, 733.09; (ICD-10) M81.0, M81.6, M81.8		

ADT, androgen deprivation therapy; DXA, dual-energy X-ray absorptiometry; HCPCS, Healthcare Common Procedure Coding System.

© 2022 Suarez-Almazor ME et al. *JAMA Network Open*.

eTable 2. Codes Used to Identify Fractures

Description	ICD-9 Diagnosis Codes	ICD-10 Diagnosis Codes
Skull fracture	800 - 804	S02.0XXA,S06.9X3A,S06.9X4A,S02.0XXB,S02.101A,S02.102A,S02.109A,S02.110A,S02.111A,S02.112A,S02.113A,S02.118A,S02.119A,S02.11AA,S02.11BA,S02.11CA,S02.11DA,S02.11EA,S02.11FA,S02.11GA,S02.11HA,S02.19XA,S02.101B,S02.102B,S02.109B,S02.110B,S02.111B,S02.112B,S02.113B,S02.118B,S02.119B,S02.11AB,S02.11BB,S02.11CB,S02.11DB,S02.11EB,S02.11FB,S02.11GB,S02.11HB,S02.19XB,S02.2XXA,S02.2XXB,S02.609A,S02.69XA,S02.610A,S02.611A,S02.612A,S02.620A,S02.621A,S02.622A,S02.630A,S02.631A,S02.632A,S02.640A,S02.641A,S02.642A,S02.650A,S02.651A,S02.652A,S02.66XA,S02.670A,S02.671A,S02.672A,S02.600A,S02.601A,S02.602A,S02.609B,S02.69XB,S02.610B,S02.611B,S02.612B,S02.620B,S02.621B,S02.622B,S02.630B,S02.631B,S02.632B,S02.640B,S02.641B,S02.642B,S02.650B,S02.651B,S02.652B,S02.66XB,S02.670B,S02.671B,S02.672B,S02.600B,S02.601B,S02.602B,S02.400A,S02.401A,S02.402A,S02.40AA,S02.40BA,S02.40CA,S02.40DA,S02.40EA,S02.40FA,S02.411A,S02.412A,S02.413A,S02.400B,S02.401B,S02.402B,S02.40AB,S02.40BB,S02.40CB,S02.40DB,S02.40EB,S02.40FB,S02.411B,S02.412B,S02.413B,S02.30XA,S02.31XA,S02.32XA,S02.30XB,S02.31XB,S02.32XB,S02.42XA,S02.80XA,S02.81XA,S02.82XA,S02.92XA,S02.42XB,S02.80XB,S02.81XB,S02.82XB,S02.92XB,S02.91XA,S02.91XB
Spine fracture	805 - 806	S12.9XXA,S12.000A,S12.001A,S12.01XA,S12.02XA,S12.030A,S12.031A,S12.040A,S12.041A,S12.090A,S12.091A,S12.100A,S12.101A,S12.110A,S12.111A,S12.112A,S12.120A,S12.121A,S12.130A,S12.131A,S12.14XA,S12.150A,S12.151A,S12.190A,S12.191A,S12.200A,S12.201A,S12.230A,S12.231A,S12.24XA,S12.250A,S12.251A,S12.290A,S12.291A,S12.300A,S12.301A,S12.330A,S12.331A,S12.34XA,S12.350A,S12.351A,S12.390A,S12.391A,S12.400A,S12.401A,S12.430A,S12.431A,S12.44XA,S12.450A,S12.451A,S12.490A,S12.491A,S12.500A,S12.501A,S12.530A,S12.531A,S12.54XA,S12.550A,S12.551A,S12.590A,S12.591A,S12.600A,S12.601A,S12.630A,S12.631A,S12.64XA,S12.650A,S12.651A,S12.690A,S12.691A,S12.000B,S12.001B,S12.01XB,S12.02XB,S12.030B,S12.031B,S12.040B,S12.041B,S12.090B,S12.091B,S12.200B,S12.201B,S12.230B,S12.231B,S12.24XB,S12.250B,S12.251B,S12.290B,S12.291B,S12.300B,S12.301B,S12.330B,S12.331B,S12.34XB,S12.350B,S12.351B,S12.390B,S12.391B,S12.400B,S12.401B,S12.430B,S12.431B,S12.44XB,S12.450B,S12.451B,S12.490B,S12.491B,S12.500B,S12.501B,S12.530B,S12.531B,S12.54XB,S12.550B,S12.551B,S12.590B,S12.591B,S12.600B,S12.601B,S12.630B,S12.631B,S12.64XB,S12.650B,S12.651B,S12.690B,S12.691B,S22.000A,S22.001A,S22.002A,S22.008A,S22.009A,S22.010A,S22.011A,S22.012A,S22.018A,S22.019A,S22.020A,S22.021A,S22.022A,S22.028A,S22.029A,S22.030A,S22.031A,S22.032A,S22.038A,S22.039A,S22.040A,S22.041A,S22.042A,S22.048A,S22.049A,S22.050A,S22.051A,S22.052A,S22.058A,S22.059A,S22.060A,S22.061A,S22.062A,S22.068A,S22.069A,S22.070A,S22.071A,S22.072A,S22.078A,S22.079A,S22.080A,S22.081A,S22.082A,S22.088

© 2022 Suarez-Almazor ME et al. *JAMA Network Open*.

		A,S22.089A,S22.000B,S22.001B,S22.002B,S22.008B,S22.009B,S22.010B,S22.011B,S22.012B,S22.018B,S22.019B,S22.020B,S22.021B,S22.022B,S22.028B,S22.029B,S22.030B,S22.031B,S22.032B,S22.038B,S22.039B,S22.040B,S22.041B,S22.042B,S22.048B,S22.049B,S22.050B,S22.051B,S22.052B,S22.058B,S22.059B,S22.060B,S22.061B,S22.062B,S22.068B,S22.069B,S22.070B,S22.071B,S22.072B,S22.078B,S22.079B,S22.080B,S22.081B,S22.082B,S22.088B,S22.089B,S32.000A,S32.001A,S32.002A,S32.008A,S32.009A,S32.010A,S32.011A,S32.012A,S32.018A,S32.019A,S32.020A,S32.021A,S32.022A,S32.028A,S32.029A,S32.030A,S32.031A,S32.032A,S32.038A,S32.039A,S32.040A,S32.041A,S32.042A,S32.048A,S32.049A,S32.050A,S32.051A,S32.052A,S32.058A,S32.059A,S32.000B,S32.001B,S32.002B,S32.008B,S32.009B,S32.010B,S32.011B,S32.012B,S32.018B,S32.019B,S32.020B,S32.021B,S32.022B,S32.028B,S32.029B,S32.030B,S32.031B,S32.032B,S32.038B,S32.039B,S32.040B,S32.041B,S32.042B,S32.048B,S32.049B,S32.050B,S32.051B,S32.052B,S32.058B,S32.059B,S32.10XA,S32.110A,S32.111A,S32.112A,S32.119A,S32.120A,S32.121A,S32.122A,S32.129A,S32.130A,S32.131A,S32.132A,S32.139A,S32.14XA,S32.15XA,S32.16XA,S32.17XA,S32.19XA,S32.2XXA,S32.10XB,S32.110B,S32.111B,S32.112B,S32.119B,S32.120B,S32.121B,S32.122B,S32.129B,S32.130B,S32.131B,S32.132B,S32.139B,S32.14XB,S32.15XB,S32.16XB,S32.17XB,S32.19XB,S32.2XXB,S14.101A,S14.102A,S14.103A,S14.104A,S14.109A,S14.111A,S14.112A,S14.113A,S14.114A,S14.131A,S14.132A,S14.133A,S14.134A,S14.121A,S14.122A,S14.123A,S14.124A,S14.151A,S14.152A,S14.153A,S14.154A,S14.105A,S14.106A,S14.107A,S14.115A,S14.116A,S14.117A,S14.135A,S14.136A,S14.137A,S14.125A,S14.126A,S14.127A,S14.155A,S14.156A,S14.157A,S24.101A,S24.102A,S24.111A,S24.112A,S24.131A,S24.132A,S24.151A,S24.152A,S22.009A,S22.069A,S22.079A,S22.089A,S24.103A,S24.104A,S24.113A,S24.114A,S24.133A,S24.134A,S24.153A,S24.154A,S34.101A,S34.102A,S34.103A,S34.104A,S34.105A,S34.109A,S34.111A,S34.112A,S34.113A,S34.114A,S34.115A,S34.119A,S34.121A,S34.122A,S34.123A,S34.124A,S34.125A,S34.129A,S34.139A,S34.3XXA,S34.131A,S34.132A,S22.39XA
Rib fracture	807.1 - 807.4	S22.39XA,S22.31XA,S22.32XA,S22.39XA,S22.41XA,S22.42XA,S22.43XA,S22.49XA,S22.39XB,S22.31XB,S22.32XB,S22.39XB,S22.41XB,S22.42XB,S22.43XB,S22.49XB,S22.20XA,S22.21XA,S22.22XA,S22.23XA,S22.24XA,S22.20XB,S22.21XB,S22.22XB,S22.23XB,S22.24XB,S22.5XXA,S22.5XXB
Pelvic fracture	808	S32.401A,S32.402A,S32.409A,S32.411A,S32.412A,S32.413A,S32.414A,S32.415A,S32.416A,S32.421A,S32.422A,S32.423A,S32.424A,S32.425A,S32.426A,S32.431A,S32.432A,S32.433A,S32.434A,S32.435A,S32.436A,S32.441A,S32.442A,S32.443A,S32.444A,S32.445A,S32.446A,S32.451A,S32.452A,S32.453A,S32.454A,S32.455A,S32.456A,S32.461A,S32.462A,S32.463A,S32.464A,S32.465A,S32.466A,S32.471A,S32.472A,S32.473A,S32.474A,S32.475A,S32.476A,S32.481A,S32.482A,S32.483A,S32.484A,S32.485A,S32.401B,S32.402B,S32.409B,S32.411B,S32.412B,S32.413B,S32.414B,S32.415B,S32.416B,S32.421B,S32.422B,S32.423B,S32.424B,S32.425B,S32.426B,S32.431B,S32.432B,S32.433B,S32.434B,S32.435B,S32.436B,S32.441B,S32.442B,S32.443B,S32.444B,S32.445B,S32.446B,S32.451B,S32.452B,S32.453B,S32.454B,S32.455B,S32.456B,S32.461B,S32.462B,S32.463B,S32.464B,S32.465B,S32.4

© 2022 Suarez-Almazor ME et al. *JAMA Network Open*.

		66B,S32.471B,S32.472B,S32.473B,S32.474B,S32.475B,S32.476B,S32.481B,S32.482B,S32.483B,S32.484B,S32.485B,S32.501A,S32.502A,S32.509A,S32.511A,S32.512A,S32.519A,S32.591A,S32.592A,S32.599A,S32.501B,S32.502B,S32. 509B,S32.511B,S32.512B,S32.519B,S32.591B,S32.592B,S32. 599B,S32.301A,S32.302A,S32.309A,S32.311A,S32.312A,S32.313A,S32.314A,S32.315A,S32.316A,S32.391A,S32.392A,S32.399A,S32.601A,S32.602A,S32.609A,S32.611A,S32.612A,S32.613A,S32.614A,S32.615A,S32.616A,S32.691A,S32.692A,S32.699A,S32.810A,S32.811A,S32.82XA,S32.89XA,S32.9XXA,S32.301B,S32.302B,S32.309B,S32.311B,S32.312B,S32.313B,S32.314B,S32.315B,S32.316B,S32.391B,S32.392B,S32.399B,S32.601B,S32.602B,S32.609B,S32.611B,S32.612B,S32.613B,S32.614B,S32.615B,S32.616B,S32. 691B,S32.692B,S32.699B,S32.810B,S32.811B,S32.82XB,S32.89XB,S32.9XXB
Upper arm fracture	810 - 812	S42.001A,S42.002A,S42.009A,S42.011A,S42.012A,S42.013A,S42.014A,S42.015A,S42.016A,S42.017A,S42.018A,S42.019A,S42.021A,S42.022A,S42.023A,S42.024A,S42.025A,S42.026A,S42.031A,S42.032A,S42.033A,S42.034A,S42.035A,S42.036A,S42.001B,S42.002B,S42.009B,S42.011B,S42.012B,S42.013B,S42.014B,S42.015B,S42.016B,S42.017B,S42.018B,S42.019B,S42.021B,S42.022B,S42.023B,S42.024B,S42.025B,S42.026B,S42.031B,S42.032B,S42.033B,S42.034B,S42.035B,S42.036B,S42.101A,S42.102A,S42.109A,S42.121A,S42.122A,S42.123A,S42.124A,S42.125A,S42.126A,S42.131A,S42.132A,S42.133A,S42.134A,S42.135A,S42.136A,S42.141A,S42.142A,S42.143A,S42.144A,S42.145A,S42.146A,S42.151A,S42.152A,S42.153A,S42.154A,S42.155A,S42.156A,S42.111A,S42.112A,S42.113A,S42.114A,S42.115A,S42.116A,S42.191A,S42.192A,S42.199A,S42.101B,S42.102B,S42.109B,S42.121B,S42.122B,S42.123B,S42.124B,S42.125B,S42.126B,S42.131B,S42.132B,S42.133B,S42.134B,S42.135B,S42.136B,S42.141B,S42.142B,S42.143B,S42.144B,S42.145B,S42.146B,S42.151B,S42.152B,S42.153B,S42.154B,S42. 155B,S42.156B,S42.111B,S42.112B,S42.113B,S42.114B,S42.115B,S42.116B,S42. 191B,S42.192B,S42.199B,S42.201A,S42.202A,S42.209A,S42.211A,S42.212A,S42.213A,S42.214A,S42.215A,S42.216A,S42.221A,S42.222A,S42.223A,S42.224A,S42.225A,S42.226A,S42.231A,S42.232A,S42.239A,S42.241A,S42.242A,S42.249A,S42.291A,S42.292A,S42.293A,S42.294A,S42.295A,S42.296A,S42.251A,S42.252A,S42.253A,S42.254A,S42.255A,S42.256A,S42.261A,S42.262A,S42.263A,S42.264A,S42.265A,S42.266A,S42.271A,S42.272A,S42.279A,S49.001A,S49.002A,S49.009A,S49.011A,S49.012A,S49.019A,S49.021A,S49.022A,S49.029A,S49.031A,S49.032A,S49.039A,S49.041A,S49.042A,S49.049A,S49.091A,S49.092A,S49.099A,S42.201B,S42.202B,S42.209B,S42.211B,S42.212B,S42.213B,S42.214B,S42.215B,S42.216B,S42.221B,S42.222B,S42.223B,S42.224B,S42.225B,S42.226B,S42.231B,S42.232B,S42.239B,S42.241B,S42.242B,S42. 249B,S42.291B,S42.292B,S42.293B,S42.294B,S42.295B,S42. 296B,S42.251B,S42.252B,S42.253B,S42.254B,S42.255B,S42.256B,S42. 261B,S42.262B,S42.263B,S42.264B,S42.265B,S42.266B,S42.301A,S42.302A,S42.309A,S42.90XA,S42.91XA,S42.92XA,S42.311A,S42.312A,S42.319A,S42.321A,S42.322A,S42.323A,S42.324A,S42.325A,S42.326A,S42.331A,S42.332A,S42.333A,S42.334A,S42.335A,S42.336A,S42.341A,S42.342A,S42.343A,S42.344A,S42.345A,S42.346A,S42.351A,S42.352A,S42.353A,S42.354A,S42.355A,S42.356A,S42.361A,S42.362A,S42.363A,S42.364A,S42.365A,S42.366A,S42.391A,S42.392A,S42.399A,S42.301B,S42.302B,S42.309B,S42.90XB,S42.9

© 2022 Suarez-Almazor ME et al. *JAMA Network Open*.

		1XB,S42.92XB,S42.321B,S42.322B,S42.323B,S42.324B,S42.325B,S42.326B,S42.331B,S42.332B,S42.333B,S42.334B,S42.335B,S42.336B,S42.341B,S42.342B,S42.343B,S42.344B,S42.345B,S42.346B,S42.351B,S42.352B,S42.353B,S42.354B,S42.355B,S42.356B,S42.361B,S42.362B,S42.363B,S42.364B,S42.365B,S42.366B,S42.391B,S42.392B,S42.399B,S42.401A,S42.402A,S42.409A,S42.411A,S42.412A,S42.413A,S42.414A,S42.415A,S42.416A,S42.421A,S42.422A,S42.423A,S42.424A,S42.425A,S42.426A,S42.431A,S42.432A,S42.433A,S42.434A,S42.435A,S42.436A,S42.451A,S42.452A,S42.453A,S42.454A,S42.455A,S42.456A,S42.441A,S42.442A,S42.443A,S42.444A,S42.445A,S42.446A,S42.447A,S42.448A,S42.449A,S42.461A,S42.462A,S42.463A,S42.464A,S42.465A,S42.466A,S42.471A,S42.472A,S42.473A,S42.474A,S42.475A,S42.476A,S49.101A,S49.102A,S49.109A,S49.111A,S49.112A,S49.119A,S49.121A,S49.122A,S49.129A,S49.131A,S49.132A,S49.139A,S49.141A,S49.142A,S49.149A,S49.191A,S49.192A,S49.199A,S42.481A,S42.482A,S42.489A,S42.491A,S42.492A,S42.493A,S42.494A,S42.495A,S42.496A,S42.401B,S42.402B,S42.409B,S42.411B,S42.412B,S42.413B,S42.414B,S42.415B,S42.416B,S42.421B,S42.422B,S42.423B,S42.424B,S42.425B,S42.426B,S42.431B,S42.432B,S42.433B,S42.434B,S42.435B,S42.436B,S42.451B,S42.452B,S42.453B,S42.454B,S42.455B,S42.456B,S42.441B,S42.442B,S42.443B,S42.444B,S42.445B,S42.446B,S42.447B,S42.448B,S42.449B,S42.461B,S42.462B,S42.463B,S42.464B,S42.465B,S42.466B,S42.471B,S42.472B,S42.473B,S42.474B,S42.475B,S42.476B,S42.491B,S42.492B,S42.493B,S42.494B,S42.495B,S42.496B
Lower arm fracture	813	S52.001A,S52.002A,S52.009A,S52.101A,S52.102A,S52.109A,S52.90XA,S52.021A,S52.022A,S52.023A,S52.024A,S52.025A,S52.026A,S52.031A,S52.032A,S52.033A,S52.034A,S52.035A,S52.036A,S52.041A,S52.042A,S52.043A,S52.044A,S52.045A,S52.046A,S52.271A,S52.272A,S52.279A,S52.001A,S52.002A,S52.009A,S52.091A,S52.092A,S52.099A,S52.121A,S52.122A,S52.123A,S52.124A,S52.125A,S52.126A,S52.131A,S52.132A,S52.133A,S52.134A,S52.135A,S52.136A,S52.101A,S52.102A,S52.109A,S52.181A,S52.182A,S52.189A,S59.101A,S59.102A,S59.109A,S59.111A,S59.112A,S59.119A,S59.121A,S59.122A,S59.129A,S59.131A,S59.132A,S59.139A,S59.141A,S59.142A,S59.149A,S59.191A,S59.192A,S59.199A,S52.90XB,S52.90XC,S52.021B,S52.021C,S52.022B,S52.022C,S52.023B,S52.023C,S52.024B,S52.024C,S52.025B,S52.025C,S52.026B,S52.026C,S52.031B,S52.031C,S52.032B,S52.032C,S52.033B,S52.033C,S52.034B,S52.034C,S52.035B,S52.035C,S52.036B,S52.036C,S52.041B,S52.041C,S52.042B,S52.042C,S52.043B,S52.043C,S52.044B,S52.044C,S52.045B,S52.045C,S52.046B,S52.046C,S52.271B,S52.271C,S52.272B,S52.272C,S52.279B,S52.279C,S52.001B,S52.001C,S52.002B,S52.002C,S52.009B,S52.009C,S52.091B,S52.091C,S52.092B,S52.092C,S52.099B,S52.099C,S52.121B,S52.121C,S52.122B,S52.122C,S52.123B,S52.123C,S52.124B,S52.124C,S52.125B,S52.125C,S52.126B,S52.126C,S52.131B,S52.131C,S52.132B,S52.132C,S52.133B,S52.133C,S52.134B,S52.134C,S52.135B,S52.135C,S52.136B,S52.136C,S52.101B,S52.101C,S52.102B,S52.102C,S52.109B,S52.109C,S52.181B,S52.181C,S52.182B,S52.182C,S52.189B,S52.189C,S52.201A,S52.202A,S52.209A,S52.301A,S52.302A,S52.309A,S52.201A,S52.202A,S52.209A,S52.211A,S52.212A,S52.219A,S52.221A,S52.222A,S52.223A,S52.224A,S52.225A,S52.226A,S52.231A,S52.232A,S52.233A,S52.234A,S52.235A,S5

© 2022 Suarez-Almazor ME et al. *JAMA Network Open*.

		2.236A,S52.241A,S52.242A,S52.243A,S52.244A,S52.245A,S52.246A,S52.251A,S52.252A,S52.253A,S52.254A,S52.255A,S52.256A,S52.261A,S52.262A,S52.263A,S52.264A,S52.265A,S52.266A,S52.281A,S52.282A,S52.283A,S52.291A,S52.292A,S52.299A,S52.201B,S52.201C,S52.202B,S52.202C,S52.209B,S52.209C,S52.301B,S52.301C,S52.302B,S52.302C,S52.309B,S52.309C,S52.301B,S52.301C,S52.302B,S52.302C,S52.309B,S52.309C,S52.321B,S52.321C,S52.322B,S52.322C,S52.323B,S52.323C,S52.324B,S52.324C,S52.325B,S52.325C,S52.326B,S52.326C,S52.331B,S52.331C,S52.332B,S52.332C,S52.333B,S52.333C,S52.334B,S52.334C,S52.335B,S52.335C,S52.336B,S52.336C,S52.341B,S52.341C,S52.342B,S52.342C,S52.343B,S52.343C,S52.344B,S52.344C,S52.345B,S52.345C,S52.346B,S52.346C,S52.351B,S52.351C,S52.352B,S52.352C,S52.353B,S52.353C,S52.354B,S52.354C,S52.201B,S52.201C,S52.202B,S52.202C,S52.209B,S52.209C,S52.221B,S52.221C,S52.222B,S52.222C,S52.223B,S52.223C,S52.224B,S52.224C,S52.225B,S52.225C,S52.226B,S52.226C,S52.231B,S52.231C,S52.232B,S52.232C,S52.233B,S52.233C,S52.234B,S52.234C,S52.235B,S52.235C,S52.236B,S52.236C,S52.241B,S52.241C,S52.242B,S52.242C,S52.243B,S52.243C,S52.244B,S52.244C,S52.245B,S52.245C,S52.246B,S52.246C,S52.251B,S52.251C,S52.252B,S52.252C,S52.253B,S52.253C,S52.254B,S52.254C,S52.531A,S52.532A,S52.539A,S52.541A,S52.542A,S52.549A,S52.501A,S52.502A,S52.509A,S52.511A,S52.512A,S52.513A,S52.514A,S52.515A,S52.516A,S52.551A,S52.552A,S52.559A,S52.561A,S52.562A,S52.569A,S52.571A,S52.572A,S52.579A,S52.591A,S52.592A,S52.599A,S59.201A,S59.202A,S59.209A,S59.211A,S59.212A,S59.219A,S59.221A,S59.222A,S59.229A,S59.231A,S59.232A,S59.239A,S59.241A,S59.242A,S59.249A,S59.291A,S59.292A,S59.299A,S52.601A,S52.602A,S52.609A,S52.611A,S52.612A,S52.613A,S52.614A,S52.615A,S52.616A,S52.691A,S52.692A,S52.699A,S59.001A,S59.002A,S59.009A,S59.011A,S59.012A,S59.019A,S59.021A,S59.022A,S59.029A,S59.031A,S59.032A,S59.039A,S59.041A,S59.042A,S59.049A,S59.091A,S59.092A,S59.099A,S52.111A,S52.112A,S52.119A,S52.521A,S52.522A,S52.529A,S52.011A,S52.012A,S52.019A,S52.621A,S52.622A,S52.629A,S52.531B,S52.531C,S52.532B,S52.532C,S52.539B,S52.539C,S52.501B,S52.501C,S52.502B,S52.502C,S52.509B,S52.509C,S52.511B,S52.511C,S52.512B,S52.512C,S52.513B,S52.513C,S52.514B,S52.514C,S52.515B,S52.515C,S52.516B,S52.516C,S52.541B,S52.541C,S52.542B,S52.542C,S52.549B,S52.549C,S52.551B,S52.551C,S52.552B,S52.552C,S52.559B,S52.559C,S52.561B,S52.561C,S52.562B,S52.562C,S52.569B,S52.569C,S52.571B,S52.571C,S52.572B,S52.572C,S52.579B,S52.579C,S52.591B,S52.591C,S52.592B,S52.592C,S52.599B,S52.599C,S52.601B,S52.601C,S52.602B,S52.602C,S52.609B,S52.609C,S52.611B,S52.611C,S52.612B,S52.612C,S52.613B,S52.613C,S52.614B,S52.614C,S52.615B,S52.615C,S52.616B,S52.616C,S52.691B,S52.691C,S52.692B,S52.692C,S52.699B,S52.699C,S52.91XA,S52.92XA,S52.91XB,S52.91XC,S52.92XB,S52.92XC
Hand fracture	814 - 817	S62.90XA,S62.90XB
Hip fracture	820	S72.011A,S72.012A,S72.019A,S72.021A,S72.022A,S72.023A,S72.024A,S72.025A,S72.026A,S79.001A,S79.002A,S79.009A,S79.011A,S79.012A,S79.019A,S79.091A,

© 2022 Suarez-Almazor ME et al. *JAMA Network Open*.

		S79.092A,S79.099A,S72.031A,S72.032A,S72.033A,S72.034A,S72.035A,S72.036A,S72.041A,S72.042A,S72.043A,S72.044A,S72.045A,S72.046A,S72.051A,S72.052A,S72.059A,S72.061A,S72.062A,S72.063A,S72.064A,S72.065A,S72.066A,S72.091A,S72.092A,S72.099A,S72.011B,S72.011C,S72.012B,S72.012C,S72.019B,S72.019C,S72.021B,S72.021C,S72.022B,S72.022C,S72.023B,S72.023C,S72.024B,S72.024C,S72.025B,S72.025C,S72.026B,S72.026C,S72.031B,S72.031C,S72.032B,S72.032C,S72.033B,S72.033C,S72.034B,S72.034C,S72.035B,S72.035C,S72.036B,S72.036C,S72.041B,S72.041C,S72.042B,S72.042C,S72.043B,S72.043C,S72.044B,S72.044C,S72.045B,S72.045C,S72.046B,S72.046C,S72.051B,S72.051C,S72.052B,S72.052C,S72.059B,S72.059C,S72.061B,S72.061C,S72.062B,S72.062C,S72.063B,S72.063C,S72.064B,S72.064C,S72.065B,S72.065C,S72.066B,S72.066C,S72.091B,S72.091C,S72.092B,S72.092C,S72.099B,S72.099C,S72.101A,S72.102A,S72.109A,S72.111A,S72.112A,S72.113A,S72.114A,S72.115A,S72.116A,S72.121A,S72.122A,S72.123A,S72.124A,S72.125A,S72.126A,S72.131A,S72.132A,S72.133A,S72.134A,S72.135A,S72.136A,S72.141A,S72.142A,S72.143A,S72.144A,S72.145A,S72.146A,S72.21XA,S72.22XA,S72.23XA,S72.24XA,S72.25XA,S72.26XA,S72.101B,S72.101C,S72.102B,S72.102C,S72.109B,S72.109C,S72.111B,S72.111C,S72.112B,S72.112C,S72.113B,S72.113C,S72.114B,S72.114C,S72.115B,S72.115C,S72.116B,S72.116C,S72.121B,S72.121C,S72.122B,S72.122C,S72.123B,S72.123C,S72.124B,S72.124C,S72.125B,S72.125C,S72.126B,S72.126C,S72.131B,S72.131C,S72.132B,S72.132C,S72.133B,S72.133C,S72.134B,S72.134C,S72.135B,S72.135C,S72.136B,S72.136C,S72.141B,S72.141C,S72.142B,S72.142C,S72.143B,S72.143C,S72.144B,S72.144C,S72.145B,S72.145C,S72.146B,S72.146C,S72.21XB,S72.21XC,S72.22XB,S72.22XC,S72.23XB,S72.23XC,S72.24XB,S72.24XC,S72.25XB,S72.25XC,S72.26XB,S72.26XC,S72.001A,S72.002A,S72.009A,S72.001B,S72.001C,S72.002B,S72.002C,S72.009B,S72.009C
Other femur fracture	821	S72.8X1A,S72.8X2A,S72.8X9A,S72.90XA,S72.91XA,S72.92XA,S72.301A,S72.302A,S72.309A,S72.321A,S72.322A,S72.323A,S72.324A,S72.325A,S72.326A,S72.331A,S72.332A,S72.333A,S72.334A,S72.335A,S72.336A,S72.341A,S72.342A,S72.343A,S72.344A,S72.345A,S72.346A,S72.351A,S72.352A,S72.353A,S72.354A,S72.355A,S72.356A,S72.361A,S72.362A,S72.363A,S72.364A,S72.365A,S72.366A,S72.391A,S72.392A,S72.399A,S72.8X1B,S72.8X1C,S72.8X2B,S72.8X2C,S72.8X9B,S72.8X9C,S72.90XB,S72.90XC,S72.91XB,S72.91XC,S72.92XB,S72.92XC,S72.301B,S72.301C,S72.302B,S72.302C,S72.309B,S72.309C,S72.321B,S72.321C,S72.322B,S72.322C,S72.323B,S72.323C,S72.324B,S72.324C,S72.325B,S72.325C,S72.326B,S72.326C,S72.331B,S72.331C,S72.332B,S72.332C,S72.333B,S72.333C,S72.334B,S72.334C,S72.335B,S72.335C,S72.336B,S72.336C,S72.341B,S72.341C,S72.342B,S72.342C,S72.343B,S72.343C,S72.344B,S72.344C,S72.345B,S72.345C,S72.346B,S72.346C,S72.351B,S72.351C,S72.352B,S72.352C,S72.353B,S72.353C,S72.354B,S72.354C,S72.401A,S72.402A,S72.409A,S72.411A,S72.412A,S72.413A,S72.414A,S72.415A,S72.416A,S72.421A,S72.422A,S72.423A,S72.424A,S72.425A,S72.426A,S72.431A,S72.432A,S72.433A,S72.434A,S72.435A,S72.436A,S72.441A,S72.442A,S72.443A,S72.444A,S72.445A,S72.446A,S79.101A,S79.102A,S79.109A,S79.111A,S79.112A,S79.119A,S79.121A,S79.122A,S79.129A,S79.131A,S79.132A,S79.139A,S79.141A,S79.142A,S79.149A,S79.191A,S79.192A,S79.199A,S72.451A,S72.452A,S72.453A,S72.454A,

© 2022 Suarez-Almazor ME et al. *JAMA Network Open*.

		S72.455A,S72.456A,S72.461A,S72.462A,S72.463A,S72.464A,S72.465A,S72.466A,S72.471A,S72.472A,S72.479A,S72.491A,S72.492A,S72.499A,S72.401B,S72.401C,S72.402B,S72.402C,S72.409B,S72.409C,S72.411B,S72.411C,S72.412B,S72.412C,S72.413B,S72.413C,S72.414B,S72.414C,S72.415B,S72.415C,S72.416B,S72.416C,S72.421B,S72.421C,S72.422B,S72.422C,S72.423B,S72.423C,S72.424B,S72.424C,S72.425B,S72.425C,S72.426B,S72.426C,S72.431B,S72.431C,S72.432B,S72.432C,S72.433B,S72.433C,S72.434B,S72.434C,S72.435B,S72.435C,S72.436B,S72.436C,S72.441B,S72.441C,S72.442B,S72.442C,S72.443B,S72.443C,S72.444B,S72.444C,S72.445B,S72.445C,S72.446B,S72.446C,S72.491B,S72.491C,S72.492B,S72.492C,S72.499B,S72.499C
Lower leg fracture	822 - 824	S82.001A,S82.002A,S82.009A,S82.011A,S82.012A,S82.013A,S82.014A,S82.015A,S82.016A,S82.021A,S82.022A,S82.023A,S82.024A,S82.025A,S82.026A,S82.031A,S82.032A,S82.033A,S82.034A,S82.035A,S82.036A,S82.041A,S82.042A,S82.043A,S82.044A,S82.045A,S82.046A,S82.091A,S82.092A,S82.099A,S82.001B,S82.001C,S82.002B,S82.002C,S82.009B,S82.009C,S82.011B,S82.011C,S82.012B,S82.012C,S82.013B,S82.013C,S82.014B,S82.014C,S82.015B,S82.015C,S82.016B,S82.016C,S82.021B,S82.021C,S82.022B,S82.022C,S82.023B,S82.023C,S82.024B,S82.024C,S82.025B,S82.025C,S82.026B,S82.026C,S82.031B,S82.031C,S82.032B,S82.032C,S82.033B,S82.033C,S82.034B,S82.034C,S82.035B,S82.035C,S82.036B,S82.036C,S82.041B,S82.041C,S82.042B,S82.042C,S82.043B,S82.043C,S82.044B,S82.044C,S82.101A,S82.102A,S82.109A,S82.111A,S82.112A,S82.113A,S82.114A,S82.115A,S82.116A,S82.121A,S82.122A,S82.123A,S82.124A,S82.125A,S82.126A,S82.131A,S82.132A,S82.133A,S82.134A,S82.135A,S82.136A,S82.141A,S82.142A,S82.143A,S82.144A,S82.145A,S82.146A,S82.151A,S82.152A,S82.153A,S82.154A,S82.155A,S82.156A,S82.191A,S82.192A,S82.199A,S89.001A,S89.002A,S89.009A,S89.011A,S89.012A,S89.019A,S89.021A,S89.022A,S89.029A,S89.031A,S89.032A,S89.039A,S89.041A,S89.042A,S82.831A,S82.832A,S82.839A,S89.201A,S89.202A,S89.209A,S89.211A,S89.212A,S89.219A,S89.221A,S89.222A,S89.229A,S89.291A,S89.292A,S89.299A,S82.101B,S82.101C,S82.102B,S82.102C,S82.109B,S82.109C,S82.111B,S82.111C,S82.112B,S82.112C,S82.113B,S82.113C,S82.114B,S82.114C,S82.115B,S82.115C,S82.116B,S82.116C,S82.121B,S82.121C,S82.122B,S82.122C,S82.123B,S82.123C,S82.124B,S82.124C,S82.125B,S82.125C,S82.126B,S82.126C,S82.131B,S82.131C,S82.132B,S82.132C,S82.133B,S82.133C,S82.134B,S82.134C,S82.135B,S82.135C,S82.136B,S82.136C,S82.141B,S82.141C,S82.142B,S82.142C,S82.143B,S82.143C,S82.144B,S82.144C,S82.831B,S82.831C,S82.832B,S82.832C,S82.839B,S82.839C,S82.201A,S82.202A,S82.209A,S82.221A,S82.222A,S82.223A,S82.224A,S82.225A,S82.226A,S82.231A,S82.232A,S82.233A,S82.234A,S82.235A,S82.236A,S82.241A,S82.242A,S82.243A,S82.244A,S82.245A,S82.246A,S82.251A,S82.252A,S82.253A,S82.254A,S82.255A,S82.256A,S82.261A,S82.262A,S82.263A,S82.264A,S82.265A,S82.266A,S82.291A,S82.292A,S82.299A,S82.401A,S82.402A,S82.409A,S82.421A,S82.422A,S82.423A,S82.424A,S82.425A,S82.426A,S82.431A,S82.432A,S82.433A,S82.434A,S82.435A,S82.436A,S82.441A,S82.442A,S82.443A,S82.444A,S82.445A,S82.446A,S82.451A,S82.452A,S82.453A,S82.454A,S82.455A,S82.456A,S82.461A,S82.462A,S82.463A,S82.464A,S82.465A,S82.466A,S82.491A,S82.492A,S82.499A,S82.

© 2022 Suarez-Almazor ME et al. *JAMA Network Open*.

		861A,S82.862A,S82.863A,S82.864A,S82.865A,S82.866A,S82.201B,S82.201C,S82.202B,S82.202C,S82.209B,S82.209C,S82.221B,S82.221C,S82.222B,S82.222C,S82.223B,S82.223C,S82.224B,S82.224C,S82.225B,S82.225C,S82.226B,S82.226C,S82.231B,S82.231C,S82.232B,S82.232C,S82.233B,S82.233C,S82.234B,S82.234C,S82.235B,S82.235C,S82.236B,S82.236C,S82.241B,S82.241C,S82.242B,S82.242C,S82.243B,S82.243C,S82.244B,S82.244C,S82.245B,S82.245C,S82.246B,S82.246C,S82.251B,S82.251C,S82.252B,S82.252C,S82.253B,S82.253C,S82.254B,S82.254C,S82.401B,S82.401C,S82.402B,S82.402C,S82.409B,S82.409C,S82.421B,S82.421C,S82.422B,S82.422C,S82.423B,S82.423C,S82.424B,S82.424C,S82.425B,S82.425C,S82.426B,S82.426C,S82.431B,S82.431C,S82.432B,S82.432C,S82.433B,S82.433C,S82.434B,S82.434C,S82.435B,S82.435C,S82.436B,S82.436C,S82.441B,S82.441C,S82.442B,S82.442C,S82.443B,S82.443C,S82.444B,S82.444C,S82.445B,S82.445C,S82.446B,S82.446C,S82.451B,S82.451C,S82.452B,S82.452C,S82.453B,S82.453C,S82.454B,S82.454C,S82.161A,S82.162A,S82.169A,S82.311A,S82.312A,S82.319A,S82.811A,S82.812A,S82.819A,S82.821A,S82.822A,S82.829A,S82.201A,S82.202A,S82.209A,S82.201B,S82.201C,S82.202B,S82.202C,S82.209B,S82.209C
Foot fracture	825 - 826	S92.001A,S92.002A,S92.009A,S92.011A,S92.012A,S92.013A,S92.014A,S92.015A,S92.016A,S92.021A,S92.022A,S92.023A,S92.024A,S92.025A,S92.026A,S92.031A,S92.032A,S92.033A,S92.034A,S92.035A,S92.036A,S92.041A,S92.042A,S92.043A,S92.044A,S92.045A,S92.046A,S92.051A,S92.052A,S92.053A,S92.054A,S92.055A,S92.056A,S92.061A,S92.062A,S92.063A,S92.064A,S92.065A,S92.066A,S99.001A,S99.002A,S99.009A,S99.011A,S99.012A,S99.019A,S99.021A,S99.022A,S99.029A,S99.031A,S99.032A,S92.001B,S92.002B,S92.009B,S92.011B,S92.012B,S92.013B,S92.014B,S92.015B,S92.016B,S92.021B,S92.022B,S92.023B,S92.024B,S92.025B,S92.026B,S92.031B,S92.032B,S92.033B,S92.034B,S92.035B,S92.036B,S92.041B,S92.042B,S92.043B,S92.044B,S92.045B,S92.046B,S92.051B,S92.052B,S92.053B,S92.054B,S92.055B,S92.056B,S92.061B,S92.062B,S92.063B,S92.064B,S92.065B,S92.066B,S99.001B,S99.002B,S99.009B,S99.011B,S99.012B,S99.019B,S99.021B,S99.022B,S99.029B,S99.031B,S99.032B,S92.811A,S92.812A,S92.819A,S92.901A,S92.902A,S92.909A,S92.101A,S92.102A,S92.109A,S92.111A,S92.112A,S92.113A,S92.114A,S92.115A,S92.116A,S92.121A,S92.122A,S92.123A,S92.124A,S92.125A,S92.126A,S92.131A,S92.132A,S92.133A,S92.134A,S92.135A,S92.136A,S92.141A,S92.142A,S92.143A,S92.144A,S92.145A,S92.146A,S92.151A,S92.152A,S92.153A,S92.154A,S92.155A,S92.156A,S92.191A,S92.192A,S92.199A,S92.251A,S92.252A,S92.253A,S92.254A,S92.255A,S92.256A,S92.211A,S92.212A,S92.213A,S92.214A,S92.215A,S92.216A,S92.221A,S92.222A,S92.223A,S92.224A,S92.225A,S92.226A,S92.231A,S92.232A,S92.233A,S92.234A,S92.235A,S92.236A,S92.241A,S92.242A,S92.243A,S92.244A,S92.245A,S92.246A,S92.301A,S92.302A,S92.309A,S92.311A,S92.312A,S92.313A,S92.314A,S92.315A,S92.316A,S92.321A,S92.322A,S92.323A,S92.324A,S92.325A,S92.326A,S92.331A,S92.332A,S92.333A,S92.334A,S92.335A,S92.336A,S92.341A,S92.342A,S92.343A,S92.344A,S92.345A,S92.346A,S92.351A,S92.352A,S92.353A,S92.354A,S92.355A,S92.356A,S99.101A,S99.102A,S99.109A,S99.111A,S99.112A,S99.119A,S99.121A,S99.122A,S99.129A,S99.131A,S99.132A,S99.139A,S99.141A,S99.142A,S99.149A,S99.191A,S99.192A,S92.201A,S92.202A,S92.209

© 2022 Suarez-Almazor ME et al. *JAMA Network Open*.

		A,S92.811B,S92.812B,S92.819B,S92.901B,S92.902B,S92.909B,S92.101B,S92.102B,S92.109B,S92.111B,S92.112B,S92.113B,S92.114B,S92.115B,S92.116B,S92.121B,S92.122B,S92.123B,S92.124B,S92.125B,S92.126B,S92.131B,S92.132B,S92.133B,S92.134B,S92.135B,S92.136B,S92.141B,S92.142B,S92.143B,S92.144B,S92.145B,S92.146B,S92.151B,S92.152B,S92.153B,S92.154B,S92.155B,S92.156B,S92.191B,S92.192B,S92.199B,S92.251B,S92.252B,S92.253B,S92.254B,S92.255B,S92.256B,S92.211B,S92.212B,S92.213B,S92.214B,S92.215B,S92.216B,S92.221B,S92.222B,S92.223B,S92.224B,S92.225B,S92.226B,S92.231B,S92.232B,S92.233B,S92.234B,S92.235B,S92.236B,S92.241B,S92.242B,S92.243B,S92.244B,S92.245B,S92.246B,S92.301B,S92.302B,S92.309B,S92.311B,S92.312B,S92.313B,S92.314B,S92.315B,S92.316B,S92.321B,S92.322B,S92.323B,S92.324B,S92.325B,S92.326B,S92.331B,S92.332B,S92.333B,S92.334B,S92.335B,S92.336B,S92.341B,S92.342B,S92.343B,S92.344B,S92.345B,S92.346B,S92.351B,S92.352B,S92.353B,S92.354B,S92.355B,S92.356B,S99.101B,S99.102B,S99.109B,S99.111B,S99.112B,S99.119B,S99.121B,S99.122B,S99.129B,S99.131B,S99.132B,S99.139B,S99.141B,S99.142B,S99.149B,S99.191B,S99.192B,S92.201B,S92.202B,S92.209B,S92.401A,S92.402A,S92.403A,S92.404A,S92.405A,S92.406A,S92.411A,S92.412A,S92.413A,S92.414A,S92.415A,S92.416A,S92.421A,S92.422A,S92.423A,S92.424A,S92.425A,S92.426A,S92.491A,S92.492A,S92.499A,S92.501A,S92.502A,S92.503A,S92.504A,S92.505A,S92.506A,S92.511A,S92.512A,S92.513A,S92.514A,S92.515A,S92.516A,S92.521A,S92.522A,S92.523A,S92.524A,S92.525A,S92.526A,S92.531A,S92.532A,S92.533A,S92.534A,S92.535A,S92.536A,S92.591A,S92.592A,S92.599A,S92.911A,S92.912A,S92.401B,S92.402B,S92.403B,S92.404B,S92.405B,S92.406B,S92.411B,S92.412B,S92.413B,S92.414B,S92.415B,S92.416B,S92.421B,S92.422B,S92.423B,S92.424B,S92.425B,S92.426B,S92.491B,S92.492B,S92.499B,S92.501B,S92.502B,S92.503B,S92.504B,S92.505B,S92.506B,S92.511B,S92.512B,S92.513B,S92.514B,S92.515B,S92.516B,S92.521B,S92.522B,S92.523B,S92.524B,S92.525B,S92.526B,S92.531B,S92.532B,S92.533B,S92.534B,S92.535B,S92.536B,S92.591B,S92.592B,S92.599B,S92.911B,S92.912B

© 2022 Suarez-Almazor ME et al. *JAMA Network Open*.

eTable 3. List of HCPCS Codes Used to Identify Bone-Modifying Agents

**Category**	**Drug**	**HCPCS Codes**
Bisphosphonate	Ibandronate sodium	J1740
	Etidronate	J1436
	Zoledronic acid	J3489, Q2051
	Zoledronic acid (Reclast)	J3488
	Zoledronic acid (Zometa)	J3487
	Pamidronate	J2430
	Alendronate	*
	Risedronate	*
Denosumab	Denosumab (Xgeva or Prolia)	J0897, C9272
Teriparatide	Teriparatide	J3110

HCPCS, Healthcare Common Procedure Coding System.

*Generic names in Part D were used to identify alendronate and risedronate use

© 2022 Suarez-Almazor ME et al. *JAMA Network Open*.

eTable 4. Proportions of Patients With Fractures After Initial ADT According to DXA Screening (N = 53 472)

Fracture	Total (N=53,472) %	No DXA (N=49,358) %	DXA (N=4,114) %	P
Any fracture	17.5	17.3	20.2	<0.001
Major fracture (spine, upper arm, lower arm, hip, other femur)	13.2	13.0	14.9	<0.001
Fracture skull	1.8	1.8	2.1	0.25
Fracture spine	6.0	5.9	7.1	0.001
Fracture rib	1.1	1.1	1.4	0.04
Fracture pelvic	1.7	1.7	2.0	0.08
Fracture upper arm	2.8	2.8	2.8	0.88
Fracture lower arm	1.9	1.9	2.2	0.17
Fracture hand	2.6	2.6	3.0	0.08
Fracture hip	5.0	5.0	5.5	0.10
Fracture other femur	2.0	2.0	2.2	0.45
Fracture lower leg	2.4	2.3	3.1	0.003
Fracture foot	2.4	2.3	2.7	0.15

DXA, dual-energy X-ray absorptiometry.

© 2022 Suarez-Almazor ME et al. *JAMA Network Open*.

eTable 5. Multivariable Cox Proportional Hazards Model for Time to First Fracture at Any Site After Initial ADT With Adjustment for Propensity Scores (n = 53 472)[a]

Covariate	Hazard ratio	95% CI	P
DXA screening			
No	1		
Yes	0.96	0.89 to 1.04	0.32
Year of initial ADT			
2005	1		
2006	0.97	0.91 to 1.04	0.46
2007	0.95	0.88 to 1.02	0.16
2008	0.81	0.75 to 0.88	<0.001
2009	0.76	0.70 to 0.83	<0.001
2010	0.72	0.65 to 0.79	<0.001
2011	0.62	0.56 to 0.68	<0.001
2012	0.55	0.5 to 0.62	<0.001
2013	0.49	0.43 to 0.55	<0.001
2014	0.31	0.26 to 0.37	<0.001
2015	0.06	0.04 to 0.08	<0.001
SEER registry			
California	1		
Connecticut	1.01	0.91 to 1.12	0.84
Detroit	1.11	1.01 to 1.23	0.039
Georgia	1.03	0.93 to 1.13	0.61
Hawaii	1.04	0.84 to 1.28	0.74
Iowa	0.97	0.87 to 1.08	0.57
Kentucky	1.12	1.01 to 1.24	0.029
Louisiana	1.15	1.04 to 1.26	0.004
New Jersey	0.99	0.93 to 1.07	0.87
New Mexico	1.16	0.99 to 1.36	0.06
Seattle	1.01	0.91 to 1.13	0.79
Texas	1.21	1.12 to 1.30	<0.001
Utah	1.00	0.86 to 1.16	0.98
Age group (years)			
66-70	1		
71-75	1.12	1.05 to 1.20	<0.001
76-80	1.50	1.41 to 1.60	<0.001
> 80	2.18	2.04 to 2.33	<0.001
Marital status			
Married	1		
Single	1.16	1.10 to 1.23	<0.001
Race and ethnicity			
Non-Hispanic White	1		
Non-Hispanic Black	0.53	0.49 to 0.58	<0.001
Hispanic	0.79	0.72 to 0.86	<0.001
Other	0.59	0.52 to 0.66	<0.001
Stage			
Localized	1		
Regional	1.17	1.08 to 1.26	<0.001

© 2022 Suarez-Almazor ME et al. *JAMA Network Open*.

Grade			
Low	1		
High	1.11	1.05 to 1.17	<0.001
Charlson score			
0	1		
1	1.22	1.16 to 1.28	<0.001
2+	1.53	1.45 to 1.62	<0.001
ADT type			
Leuprolide only	1		
Goserelin only	1.00	0.92 to 1.09	0.93
Triptorelin only	0.92	0.84 to 1.01	0.06
Abarelix or degarelix only	0.89	0.63 to 1.26	0.51
Histrelin only	1.24	1.01 to 1.52	0.038
Nonsteroidal antiandrogen	0.75	0.58 to 0.96	0.024
2+ ADT types	1.08	1.02 to 1.14	0.005
State buy-in			
None	1		
Full/partial	1.15	1.08 to 1.23	<0.001
Osteoporosis before 1st ADT			
No	1		
Yes	1.42	0.85 to 2.40	0.18

[a]All patients with prior fractures or fractures before their last DXA claim were excluded. DXA screening was forced in the multivariable model. Adjusted variables remained in the model based on both clinical and statistical significance.

Abbreviations: CI, confidence interval; DXA, dual-energy X-ray absorptiometry; SEER, Surveillance, Epidemiology, and End Results; ADT, androgen deprivation therapy.

© 2022 Suarez-Almazor ME et al. *JAMA Network Open*.

eFigure. Data Sources and Cohort Selection

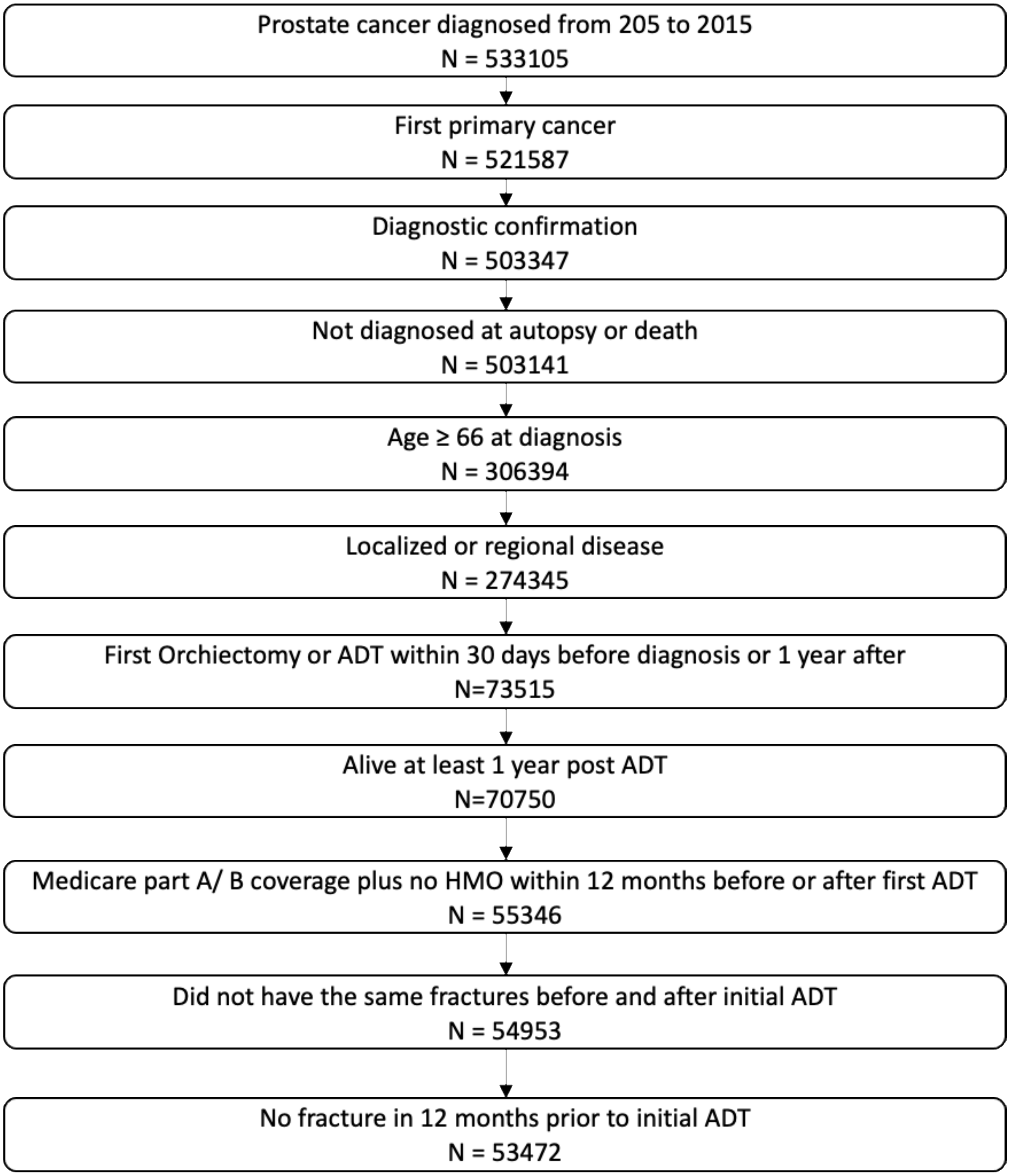

ADT, Androgen deprivation therapy; DXA, Dual Energy X-ray Absorptiometry; HMO, Health Maintenance Organization

© 2022 Suarez-Almazor ME et al. *JAMA Network Open*.
